# Bis(1,4,7-trithia­cyclo­nona­ne)nickel(II) bis­(tetra­fluorido­borate) nitro­methane disolvate

**DOI:** 10.1107/S1600536811037809

**Published:** 2011-09-30

**Authors:** John P. Lee, Gregory J. Grant, Bruce C. Noll

**Affiliations:** a Department of Chemistry, The University of Tennessee at Chattanoga, Chattanooga, TN 37403, USA; b Crystallographic Systems, Bruker AXS Inc., 5465 East Cheryl Parkway, Madison, WI 53711, USA

## Abstract

The homoleptic thio­ether title complex, [Ni(C_6_H_12_S_3_)_2_](BF_4_)_2_·2CH_3_NO_2_, shows the expeced hexa­kis­(thio­ether) octa­hedral environment around the Ni^II^ atom. It crystallized as two crystallographically independent complex cations, [Ni(9S3)_2_]^2+^ (9S3 = 1,4,7-trithia­cyclo­nona­ne), within the unit cell where each Ni^II^ lies on an inversion center. In addition to the complex cations, there are two crystallographically independent BF_4_^−^ anions present to balance the charge, and each shows disorder along a pseudo-*C*_3_ axis with ratios of 0.53 (2):0.47 (2) and 0.55 (2):0.45 (2). Two nitro­methane solvent mol­ecules per complex cation are also present in the unit cell.

## Related literature

For other related Ni^II^ complexes, see: Setzer *et al.* (1983 ▶); Blake *et al.* (1992 ▶, 2001 ▶, 2007 ▶); Nishijo *et al.* (2003 ▶, 2004 ▶). For the coordination chemistry of 1,4,7-trithia­cyclo­nonane, see: Blake & Schroder (1990 ▶); Cooper & Rawle (1990 ▶); Glass *et al.* (1980 ▶); Grant *et al.* (1991 ▶); Helm *et al.* (2005 ▶); Setzer *et al.* (1990 ▶). For related complexes that incorporate nitro­methane, see: Grant *et al.* (2005 ▶); Helm *et al.* (2006 ▶). For a description of the Cambridge Structural Database, see: Allen (2002 ▶).
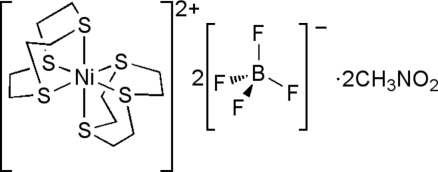


## Experimental

### 

#### Crystal data

[Ni(C_6_H_12_S_3_)_2_](BF_4_)_2_·2CH_3_NO_2_
*M* *_r_* = 715.09
Monoclinic, 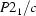

*a* = 9.1755 (18) Å
*b* = 19.825 (4) Å
*c* = 15.173 (3) Å
β = 90.88 (3)°
*V* = 2759.6 (9) Å^3^
*Z* = 4
Mo *K*α radiation
μ = 1.24 mm^−1^
*T* = 200 K
0.40 × 0.20 × 0.10 mm

#### Data collection

Bruker SMART X2S benchtop crystallographic system diffractometer
Absorption correction: multi-scan (*SADABS*; Sheldrick, 2004 ▶) *T*_min_ = 0.371, *T*_max_ = 0.941
25688 measured reflections
4889 independent reflections
3855 reflections with *I* > 2σ(*I*)
*R*_int_ = 0.054

#### Refinement

*R*[*F* ^2^ > 2σ(*F* ^2^)] = 0.037
*wR*(*F* ^2^) = 0.090
*S* = 1.05
4889 reflections
395 parameters
258 restraints
H-atom parameters constrained
Δρ_max_ = 0.56 e Å^−3^
Δρ_min_ = −0.39 e Å^−3^

### 

Data collection: *APEX2* (Bruker, 2007 ▶); cell refinement: *SAINT* (Bruker, 2007 ▶); data reduction: *SAINT*; program(s) used to solve structure: *SHELXS97* (Sheldrick, 2008 ▶); program(s) used to refine structure: *SHELXL97* (Sheldrick, 2008 ▶); molecular graphics: *SHELXTL* (Sheldrick, 2008 ▶); software used to prepare material for publication: *SHELXTL* and local programs.

## Supplementary Material

Crystal structure: contains datablock(s) I, global. DOI: 10.1107/S1600536811037809/br2171sup1.cif

Structure factors: contains datablock(s) I. DOI: 10.1107/S1600536811037809/br2171Isup2.hkl

Additional supplementary materials:  crystallographic information; 3D view; checkCIF report

## Figures and Tables

**Fig. 1.**
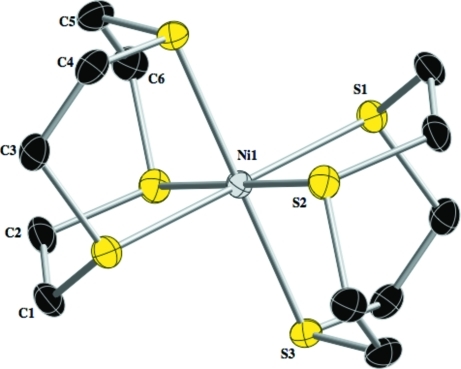
Thermal ellipsoid perspective (50% probability) showing one complex cation. Hydrogen atoms have been omitted forclarity.

**Fig. 2.**
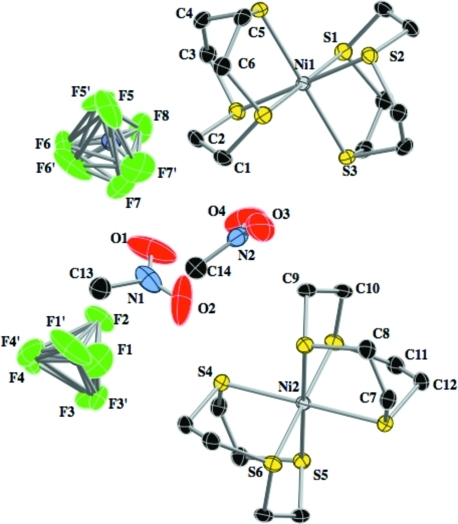
Thermal ellipsoid perspective (50% probability) showing all crystallographically independent complex cations,anions and solvate molecules. Hydrogen atoms have been omitted for clarity.

## supplementary crystallographic information

### Comment

The coordination chemistry of 1,4,7-trithiacyclononane (9S3) has been well studied both by us as well as other groups (Grant *et al.*, 1991; Helm *et al.*, 2005; Helm *et al.*, 2006; Setzer *et al.*, 1990; Setzer *et al.*, 1983; Cooper *et al.*, 1990; Blake *et al.*, 1990). The three sulfur atoms of the 9S3 ligand have been calculated to be all endodentate in the lowest energy conformation of the free ligand (Glass *et al.*, 1980). The endodentate nature of the sulfur atoms of 9S3 provides facile facial coordination to metal centers, and 9S3 has been complexed, in a bis-homoleptic fashion {*i.e.*, [*M*(9S3)_2_]^n+^}, to 26 transistion metals ions to give a total of 72 different structures in a recent search of the Cambridge Structural Database (Allen, 2002; Release with Feb. and May 2011 updates). The complex, bis(1,4,7-trithiacyclononane)nickel(II) tetrafluoroborate, has been previously synthesized and characterized, which includes a single- crystal X-ray crystal structure where the crystals were obtained from ethanol evaporation with exclusion of any solvent in the structure (Setzer *et al.*, 1983). In addition, the structure of the dication [Ni(9S3)_2_]^2+^ has been crystallographically characterized using a number of different anions (Blake *et al.*, 2007; Blake *et al.*, 2001; Nishijo *et al.*, 2003; Nishijo *et al.*, 2004; Blake *et al.*, 1992). Herein, we wish to report the structure of the coordination compound bis(1,4,7-trithiacyclononane)nickel(II)bistetrafluoroborate dinitromethane solvate to include with the previously reported [Ni(9S3)_2_]^2+^ complexes.

As can be seen in Figure 1, the title complex displays the expected hexakis (thioether) octahedral geometry around the Ni^II^ center where each 9S3 ligand is coordinated to the face of the octahedron. Interestingly, the title complex crystallizes as two crystallographically independent complex cations within the unit cell where each Ni^II^ cation lies at the inversion center (Figure 2). In addition, both the tetrafluoroborate anion and nitromethane solvate crystallize as two crystallographically independent anions and solvent molecules, respectively. Out of the seven crystallographically characterized [Ni(9S3)_2_]^2+^ complex cations this is the first example where the unit cell contains two crystallographically different complex cations. Packing within the monoclinic crystal lattice shows a face-centered array for the [Ni(9S3)_2_]^2+^ dication to give a total of four [Ni(9S3)_2_]^2+^ complex cations within the unit cell. Both the BF_4_^-^ anions and the nitromethane solvate molecules occupy four locations on the face and four locations within the unit cell to give a total of eight BF_4_^-^ anions and eight nitromethane solvate molecules. Thus, the molecular formula is [Ni(9S3)_2_][BF_4_]_2_.2CH_3_NO_2_.

Setzer *et al.* have published the structure of [Ni(9S3)_2_][BF_4_]_2_ that was crystallized from ethanol, and was solved in the same space group (*P*2_1_/*c*) and approximately the same R1 (0.03) as the title compound (Setzer *et al.*, 1983).  However, there are several differences between the previously reported structure of the title complex crystallized from ethanol *versus* that reported herein from nitromethane. Most notably is the presence of two crystallographically different complex cations. This is likely due, at least in part, to differences in crystal packing in the two different solvents. Secondly, the incorporation of solvent in the crystal lattice is unique to this system, but not unprecedented for crystal growth of similar structures from nitromethane (Helm *et al.*, 2006; Grant *et al.*, 2005).  Lastly, there is a difference in the tetrafluoroborate anions in the structures. In the original report (Setzer *et al.*, 1983), no disorder was observed in the BF_4_^-^ anions compared to the compound shown in Figure 2 where disorder in the B-F bonds along a C_3_ axis is observed. Again, this could be attributed to different crystal packing in the two different solvents; however, temperature effects cannot be discounted as data collection on the Bruker *SMART* X2S was done at 203 K.

### Experimental

Bis(1,4,7-trithiacyclononane)nickel(II) bistetrafluoroborate dinitromethane solvate was synthesized as previously reported (Setzer *et al.*, 1983). Violet needle-like crystals were obtained by slow diffusion of diethyl ether into a concentrated CH_3_NO_2_ solution of the title complex.

### Refinement

All hydrogen atoms were set to calculated geometries and allowed to refine on the positions of the parent atoms.

Structure refinement revealed disorder in the BF_4_ anions. In each case there is rotation about a B—F bond, which creates alternative sites for the remaining F atoms.  The B—F distances as well as the F^···^F distances were restrained to be equal within 0.02 Å. The model contains 174 such restraints. This disorder also manifests itself in the anisotropic thermal parameters of the F atoms.  The U_ij_ components were restrained to approximate isotropic behavior.  In all, 258 restraints were applied.

### Crystal data

**Table:**

[Ni(C_6_H_12_S_3_)_2_](BF_4_)_2_·2CH_3_NO_2_	*F*(000) = 1464
*M**_r_* = 715.09	*D*_x_ = 1.721 Mg m^−^^3^
Monoclinic, *P*2_1_/*c*	Mo *K*α radiation, λ = 0.71073 Å
Hall symbol: -P 2ybc	Cell parameters from 241 reflections
*a* = 9.1755 (18) Å	θ = 2.8–26.2°
*b* = 19.825 (4) Å	µ = 1.24 mm^−^^1^
*c* = 15.173 (3) Å	*T* = 200 K
β = 90.88 (3)°	Needle, violet
*V* = 2759.6 (9) Å^3^	0.40 × 0.20 × 0.10 mm
*Z* = 4	

### Data collection

**Table:**

Bruker SMART X2S benchtop crystallographic system diffractometer	4889 independent reflections
Radiation source: sealed tube	3855 reflections with *I* > 2σ(*I*)
graphite	*R*_int_ = 0.054
Detector resolution: 8.3330 pixels mm^-1^	θ_max_ = 25.3°, θ_min_ = 1.7°
thin–slice ω scans	*h* = −10→10
Absorption correction: multi-scan (*SADABS*; Sheldrick, 2004)	*k* = −23→23
*T*_min_ = 0.371, *T*_max_ = 0.941	*l* = −18→18
25688 measured reflections	

### Refinement

**Table:**

Refinement on *F*^2^	Primary atom site location: structure-invariant direct methods
Least-squares matrix: full	Secondary atom site location: difference Fourier map
*R*[*F*^2^ > 2σ(*F*^2^)] = 0.037	Hydrogen site location: inferred from neighbouring sites
*wR*(*F*^2^) = 0.090	H-atom parameters constrained
*S* = 1.05	*w* = 1/[σ^2^(*F*_o_^2^) + (0.0364*P*)^2^ + 1.8397*P*] where *P* = (*F*_o_^2^ + 2*F*_c_^2^)/3
4889 reflections	(Δ/σ)_max_ = 0.001
395 parameters	Δρ_max_ = 0.56 e Å^−^^3^
258 restraints	Δρ_min_ = −0.39 e Å^−^^3^

### Special details

**Table:**

Geometry. All e.s.d.'s (except the e.s.d. in the dihedral angle between two l.s. planes) are estimated using the full covariance matrix. The cell e.s.d.'s are taken into account individually in the estimation of e.s.d.'s in distances, angles and torsion angles; correlations between e.s.d.'s in cell parameters are only used when they are defined by crystal symmetry. An approximate (isotropic) treatment of cell e.s.d.'s is used for estimating e.s.d.'s involving l.s. planes.
Refinement. Refinement of *F*^2^ against ALL reflections. The weighted *R*-factor *wR* and goodness of fit *S* are based on *F*^2^, conventional *R*-factors *R* are based on *F*, with *F* set to zero for negative *F*^2^. The threshold expression of *F*^2^ > σ(*F*^2^) is used only for calculating *R*-factors(gt) *etc*. and is not relevant to the choice of reflections for refinement. *R*-factors based on *F*^2^ are statistically about twice as large as those based on *F*, and *R*- factors based on ALL data will be even larger.

### Fractional atomic coordinates and isotropic or equivalent isotropic displacement parameters (Å^2^)

**Table:**

	*x*	*y*	*z*	*U*_iso_*/*U*_eq_	Occ. (<1)
Ni1	1.0000	0.5000	0.5000	0.02711 (14)	
S1	0.74469 (9)	0.49669 (4)	0.53181 (6)	0.0376 (2)	
S2	0.96856 (9)	0.61209 (4)	0.44732 (5)	0.0370 (2)	
S3	1.03907 (9)	0.54595 (4)	0.64348 (5)	0.0355 (2)	
C1	0.6840 (4)	0.5643 (2)	0.4602 (2)	0.0458 (9)	
H1A	0.6825	0.5474	0.3988	0.055*	
H1B	0.5826	0.5760	0.4754	0.055*	
C2	0.7751 (3)	0.62848 (19)	0.4635 (2)	0.0448 (8)	
H2A	0.7623	0.6506	0.5213	0.054*	
H2B	0.7396	0.6599	0.4172	0.054*	
C3	1.0551 (4)	0.65842 (18)	0.5360 (2)	0.0447 (8)	
H3A	1.1620	0.6541	0.5303	0.054*	
H3B	1.0303	0.7068	0.5291	0.054*	
C4	1.0139 (4)	0.63624 (18)	0.6276 (2)	0.0453 (8)	
H4A	0.9106	0.6479	0.6378	0.054*	
H4B	1.0744	0.6610	0.6714	0.054*	
C5	0.8694 (4)	0.52059 (18)	0.6949 (2)	0.0421 (8)	
H5A	0.8742	0.4717	0.7076	0.051*	
H5B	0.8613	0.5445	0.7519	0.051*	
C6	0.7342 (4)	0.5343 (2)	0.6409 (2)	0.0438 (8)	
H6A	0.7205	0.5836	0.6350	0.053*	
H6B	0.6487	0.5158	0.6716	0.053*	
Ni2	0.5000	0.5000	1.0000	0.02718 (14)	
S4	0.46860 (9)	0.61205 (4)	1.05263 (5)	0.0370 (2)	
S5	0.53896 (9)	0.54594 (4)	0.85648 (5)	0.0356 (2)	
S6	0.24477 (9)	0.49668 (4)	0.96823 (6)	0.0376 (2)	
C7	0.5552 (4)	0.65834 (18)	0.9641 (2)	0.0443 (8)	
H7A	0.5306	0.7067	0.9704	0.053*	
H7B	0.6622	0.6540	0.9716	0.053*	
C8	0.5141 (4)	0.63632 (18)	0.8721 (2)	0.0443 (8)	
H8A	0.5746	0.6610	0.8294	0.053*	
H8B	0.4108	0.6481	0.8601	0.053*	
C9	0.3690 (4)	0.52056 (19)	0.8050 (2)	0.0421 (8)	
H9A	0.3584	0.5443	0.7479	0.050*	
H9B	0.3735	0.4716	0.7924	0.050*	
C10	0.2341 (4)	0.53443 (19)	0.8593 (2)	0.0436 (8)	
H10A	0.1474	0.5165	0.8276	0.052*	
H10B	0.2215	0.5838	0.8655	0.052*	
C11	0.1840 (4)	0.56433 (19)	1.0398 (2)	0.0456 (9)	
H11A	0.0821	0.5758	1.0235	0.055*	
H11B	0.1843	0.5475	1.1012	0.055*	
C12	0.2751 (4)	0.62830 (19)	1.0366 (2)	0.0452 (9)	
H12A	0.2419	0.6597	1.0829	0.054*	
H12B	0.2593	0.6505	0.9788	0.054*	
B1	0.5621 (4)	0.3228 (2)	0.3852 (3)	0.0454 (10)	
F2	0.4223 (3)	0.31428 (16)	0.3592 (2)	0.0965 (10)	
F1	0.6404 (15)	0.2690 (6)	0.3621 (9)	0.126 (5)	0.47 (2)
F3	0.5603 (13)	0.3216 (7)	0.4745 (5)	0.098 (4)	0.47 (2)
F4	0.6175 (16)	0.3788 (7)	0.3580 (12)	0.138 (7)	0.47 (2)
F1'	0.6551 (9)	0.2978 (11)	0.3282 (12)	0.161 (7)	0.53 (2)
F3'	0.5895 (16)	0.3014 (8)	0.4656 (8)	0.127 (5)	0.53 (2)
F4'	0.5896 (13)	0.3911 (4)	0.3868 (9)	0.090 (3)	0.53 (2)
B2	0.9384 (4)	0.6773 (2)	0.8853 (3)	0.0466 (10)	
F5	0.9375 (14)	0.6777 (8)	0.9741 (5)	0.102 (5)	0.45 (2)
F6	0.8808 (17)	0.6223 (7)	0.8555 (13)	0.139 (8)	0.45 (2)
F7	0.8620 (15)	0.7319 (6)	0.8613 (10)	0.130 (6)	0.45 (2)
F8	1.0777 (3)	0.68555 (16)	0.8592 (2)	0.0960 (10)	
F5'	0.9137 (15)	0.6977 (8)	0.9660 (7)	0.135 (6)	0.55 (2)
F6'	0.9095 (12)	0.6092 (4)	0.8865 (8)	0.086 (3)	0.55 (2)
F7'	0.8436 (9)	0.7031 (10)	0.8294 (12)	0.163 (7)	0.55 (2)
N1	0.5976 (5)	0.3452 (3)	0.6971 (3)	0.0804 (13)	
O1	0.4758 (5)	0.3253 (4)	0.6821 (3)	0.185 (3)	
O2	0.6242 (8)	0.3969 (3)	0.7308 (5)	0.179 (3)	
C13	0.7127 (5)	0.3000 (2)	0.6732 (3)	0.0674 (12)	
H13A	0.7133	0.2610	0.7130	0.101*	
H13B	0.8065	0.3235	0.6780	0.101*	
H13C	0.6971	0.2847	0.6124	0.101*	
N2	0.9028 (5)	0.6549 (3)	0.1971 (3)	0.0808 (13)	
O3	0.8747 (8)	0.6035 (3)	0.2312 (5)	0.184 (3)	
O4	1.0248 (5)	0.6746 (4)	0.1823 (3)	0.186 (4)	
C14	0.7875 (5)	0.7002 (2)	0.1735 (3)	0.0678 (12)	
H14A	0.6936	0.6774	0.1803	0.102*	
H14B	0.7983	0.7142	0.1120	0.102*	
H14C	0.7918	0.7399	0.2118	0.102*	

### Atomic displacement parameters (Å^2^)

**Table:**

	*U*^11^	*U*^22^	*U*^33^	*U*^12^	*U*^13^	*U*^23^
Ni1	0.0251 (3)	0.0323 (3)	0.0239 (3)	0.0019 (2)	0.0015 (2)	−0.0014 (2)
S1	0.0277 (4)	0.0460 (5)	0.0393 (5)	−0.0004 (4)	0.0041 (3)	−0.0042 (4)
S2	0.0370 (5)	0.0401 (5)	0.0340 (4)	0.0038 (4)	0.0024 (3)	0.0056 (3)
S3	0.0386 (5)	0.0403 (5)	0.0274 (4)	0.0029 (3)	−0.0026 (3)	−0.0032 (3)
C1	0.0305 (18)	0.063 (2)	0.044 (2)	0.0107 (16)	−0.0046 (14)	0.0016 (17)
C2	0.0359 (19)	0.050 (2)	0.048 (2)	0.0160 (16)	−0.0016 (15)	0.0048 (17)
C3	0.049 (2)	0.0345 (19)	0.051 (2)	−0.0017 (16)	−0.0044 (16)	0.0021 (16)
C4	0.054 (2)	0.037 (2)	0.045 (2)	−0.0006 (16)	−0.0049 (16)	−0.0117 (15)
C5	0.053 (2)	0.047 (2)	0.0269 (17)	−0.0001 (16)	0.0105 (14)	−0.0012 (14)
C6	0.042 (2)	0.052 (2)	0.0376 (19)	0.0048 (16)	0.0141 (15)	−0.0005 (16)
Ni2	0.0252 (3)	0.0323 (3)	0.0240 (3)	0.0022 (2)	0.0000 (2)	0.0015 (2)
S4	0.0371 (5)	0.0399 (5)	0.0339 (4)	0.0037 (4)	−0.0006 (3)	−0.0052 (3)
S5	0.0391 (5)	0.0402 (5)	0.0276 (4)	0.0022 (3)	0.0043 (3)	0.0032 (3)
S6	0.0276 (4)	0.0455 (5)	0.0396 (5)	−0.0010 (4)	−0.0022 (3)	0.0039 (4)
C7	0.049 (2)	0.0332 (19)	0.051 (2)	−0.0044 (16)	0.0063 (16)	−0.0011 (15)
C8	0.050 (2)	0.037 (2)	0.046 (2)	0.0029 (16)	0.0051 (16)	0.0096 (15)
C9	0.049 (2)	0.049 (2)	0.0281 (17)	0.0017 (16)	−0.0070 (14)	0.0023 (14)
C10	0.044 (2)	0.049 (2)	0.0372 (19)	0.0046 (17)	−0.0131 (14)	0.0011 (16)
C11	0.0302 (18)	0.063 (2)	0.044 (2)	0.0095 (16)	0.0066 (14)	−0.0004 (17)
C12	0.038 (2)	0.052 (2)	0.045 (2)	0.0158 (16)	0.0034 (15)	−0.0059 (17)
B1	0.040 (2)	0.049 (3)	0.047 (2)	0.0007 (19)	−0.0008 (18)	0.000 (2)
F2	0.0520 (16)	0.097 (2)	0.140 (3)	−0.0027 (15)	−0.0223 (15)	−0.0342 (19)
F1	0.130 (10)	0.091 (7)	0.159 (11)	0.067 (6)	0.022 (7)	−0.010 (6)
F3	0.129 (7)	0.122 (9)	0.045 (5)	0.035 (6)	0.016 (4)	−0.011 (5)
F4	0.123 (9)	0.145 (12)	0.145 (12)	−0.047 (9)	−0.026 (8)	0.123 (11)
F1'	0.070 (5)	0.256 (16)	0.160 (11)	−0.004 (7)	0.037 (6)	−0.144 (11)
F3'	0.138 (9)	0.120 (8)	0.123 (9)	−0.064 (7)	−0.051 (7)	0.083 (7)
F4'	0.094 (6)	0.061 (4)	0.115 (7)	−0.025 (4)	−0.009 (5)	−0.008 (5)
B2	0.042 (2)	0.050 (3)	0.047 (2)	−0.002 (2)	0.0008 (18)	−0.002 (2)
F5	0.123 (8)	0.142 (10)	0.039 (5)	0.034 (7)	−0.012 (5)	0.018 (5)
F6	0.125 (10)	0.152 (14)	0.140 (13)	−0.047 (10)	0.023 (8)	−0.120 (12)
F7	0.132 (10)	0.086 (7)	0.171 (12)	0.067 (7)	−0.030 (7)	0.003 (7)
F8	0.0526 (16)	0.098 (2)	0.138 (3)	−0.0020 (15)	0.0230 (16)	0.0343 (19)
F5'	0.151 (9)	0.131 (8)	0.123 (9)	−0.073 (7)	0.059 (7)	−0.094 (7)
F6'	0.094 (5)	0.059 (4)	0.104 (7)	−0.023 (3)	0.011 (4)	0.000 (4)
F7'	0.067 (5)	0.248 (15)	0.175 (11)	−0.005 (7)	−0.035 (5)	0.147 (11)
N1	0.066 (3)	0.100 (4)	0.077 (3)	0.028 (3)	0.022 (2)	0.043 (3)
O1	0.054 (3)	0.376 (11)	0.125 (4)	0.029 (4)	0.000 (2)	0.095 (5)
O2	0.275 (8)	0.060 (3)	0.206 (6)	0.029 (4)	0.132 (6)	0.008 (3)
C13	0.065 (3)	0.071 (3)	0.066 (3)	0.004 (2)	0.004 (2)	−0.001 (2)
N2	0.068 (3)	0.101 (4)	0.073 (3)	0.026 (3)	−0.023 (2)	−0.044 (3)
O3	0.278 (8)	0.060 (3)	0.209 (6)	0.032 (4)	−0.133 (6)	−0.008 (4)
O4	0.052 (3)	0.376 (11)	0.131 (4)	0.028 (4)	0.002 (2)	−0.097 (5)
C14	0.067 (3)	0.068 (3)	0.068 (3)	0.004 (2)	−0.003 (2)	0.000 (2)

### Geometric parameters (Å, °)

**Table:**

Ni1—S2^i^	2.3776 (9)	C7—H7A	0.9900
Ni1—S2	2.3776 (9)	C7—H7B	0.9900
Ni1—S3	2.3820 (9)	C8—H8A	0.9900
Ni1—S3^i^	2.3821 (9)	C8—H8B	0.9900
Ni1—S1^i^	2.3999 (9)	C9—C10	1.522 (5)
Ni1—S1	2.3999 (9)	C9—H9A	0.9900
S1—C1	1.808 (4)	C9—H9B	0.9900
S1—C6	1.819 (3)	C10—H10A	0.9900
S2—C3	1.803 (4)	C10—H10B	0.9900
S2—C2	1.825 (3)	C11—C12	1.520 (5)
S3—C4	1.820 (4)	C11—H11A	0.9900
S3—C5	1.822 (3)	C11—H11B	0.9900
C1—C2	1.523 (5)	C12—H12A	0.9900
C1—H1A	0.9900	C12—H12B	0.9900
C1—H1B	0.9900	B1—F4	1.291 (8)
C2—H2A	0.9900	B1—F3'	1.312 (8)
C2—H2B	0.9900	B1—F1'	1.321 (8)
C3—C4	1.512 (5)	B1—F1	1.336 (8)
C3—H3A	0.9900	B1—F2	1.347 (5)
C3—H3B	0.9900	B1—F3	1.355 (8)
C4—H4A	0.9900	B1—F4'	1.377 (8)
C4—H4B	0.9900	B2—F6	1.289 (9)
C5—C6	1.500 (5)	B2—F7'	1.310 (8)
C5—H5A	0.9900	B2—F5'	1.312 (8)
C5—H5B	0.9900	B2—F7	1.338 (8)
C6—H6A	0.9900	B2—F5	1.348 (8)
C6—H6B	0.9900	B2—F8	1.354 (5)
Ni2—S4^ii^	2.3795 (9)	B2—F6'	1.375 (8)
Ni2—S4	2.3795 (9)	N1—O2	1.170 (7)
Ni2—S6^ii^	2.3846 (10)	N1—O1	1.204 (7)
Ni2—S6	2.3846 (10)	N1—C13	1.435 (6)
Ni2—S5	2.3923 (9)	C13—H13A	0.9800
Ni2—S5^ii^	2.3923 (9)	C13—H13B	0.9800
S4—C12	1.817 (3)	C13—H13C	0.9800
S4—C7	1.819 (4)	N2—O3	1.174 (7)
S5—C9	1.805 (3)	N2—O4	1.210 (6)
S5—C8	1.822 (4)	N2—C14	1.428 (6)
S6—C10	1.815 (3)	C14—H14A	0.9800
S6—C11	1.819 (4)	C14—H14B	0.9800
C7—C8	1.505 (5)	C14—H14C	0.9800
			
S2^i^—Ni1—S2	180.000 (1)	S4^ii^—Ni2—S4	180.00 (4)
S2^i^—Ni1—S3	91.97 (3)	S4^ii^—Ni2—S6^ii^	88.28 (3)
S2—Ni1—S3	88.03 (3)	S4—Ni2—S6^ii^	91.72 (3)
S2^i^—Ni1—S3^i^	88.03 (3)	S4^ii^—Ni2—S6	91.72 (3)
S2—Ni1—S3^i^	91.97 (3)	S4—Ni2—S6	88.28 (3)
S3—Ni1—S3^i^	180.0	S6^ii^—Ni2—S6	180.00 (4)
S2^i^—Ni1—S1^i^	88.82 (3)	S4^ii^—Ni2—S5	91.68 (3)
S2—Ni1—S1^i^	91.18 (3)	S4—Ni2—S5	88.32 (3)
S3—Ni1—S1^i^	92.27 (4)	S6^ii^—Ni2—S5	90.78 (4)
S3^i^—Ni1—S1^i^	87.73 (4)	S6—Ni2—S5	89.22 (4)
S2^i^—Ni1—S1	91.18 (3)	S4^ii^—Ni2—S5^ii^	88.32 (3)
S2—Ni1—S1	88.82 (3)	S4—Ni2—S5^ii^	91.68 (3)
S3—Ni1—S1	87.73 (4)	S6^ii^—Ni2—S5^ii^	89.22 (4)
S3^i^—Ni1—S1	92.27 (4)	S6—Ni2—S5^ii^	90.78 (4)
S1^i^—Ni1—S1	180.0	S5—Ni2—S5^ii^	180.00 (4)
C1—S1—C6	102.91 (17)	C12—S4—C7	104.40 (17)
C1—S1—Ni1	98.70 (12)	C12—S4—Ni2	104.10 (12)
C6—S1—Ni1	103.71 (12)	C7—S4—Ni2	99.60 (12)
C3—S2—C2	103.14 (17)	C9—S5—C8	102.78 (17)
C3—S2—Ni1	100.11 (12)	C9—S5—Ni2	98.47 (11)
C2—S2—Ni1	103.56 (12)	C8—S5—Ni2	103.58 (11)
C4—S3—C5	102.74 (17)	C10—S6—C11	103.12 (17)
C4—S3—Ni1	103.76 (11)	C10—S6—Ni2	102.22 (12)
C5—S3—Ni1	99.66 (11)	C11—S6—Ni2	99.66 (12)
C2—C1—S1	115.8 (2)	C8—C7—S4	115.6 (2)
C2—C1—H1A	108.3	C8—C7—H7A	108.4
S1—C1—H1A	108.3	S4—C7—H7A	108.4
C2—C1—H1B	108.3	C8—C7—H7B	108.4
S1—C1—H1B	108.3	S4—C7—H7B	108.4
H1A—C1—H1B	107.4	H7A—C7—H7B	107.4
C1—C2—S2	112.4 (2)	C7—C8—S5	112.1 (2)
C1—C2—H2A	109.1	C7—C8—H8A	109.2
S2—C2—H2A	109.1	S5—C8—H8A	109.2
C1—C2—H2B	109.1	C7—C8—H8B	109.2
S2—C2—H2B	109.1	S5—C8—H8B	109.2
H2A—C2—H2B	107.9	H8A—C8—H8B	107.9
C4—C3—S2	115.1 (3)	C10—C9—S5	114.8 (2)
C4—C3—H3A	108.5	C10—C9—H9A	108.6
S2—C3—H3A	108.5	S5—C9—H9A	108.6
C4—C3—H3B	108.5	C10—C9—H9B	108.6
S2—C3—H3B	108.5	S5—C9—H9B	108.6
H3A—C3—H3B	107.5	H9A—C9—H9B	107.5
C3—C4—S3	112.0 (2)	C9—C10—S6	112.7 (2)
C3—C4—H4A	109.2	C9—C10—H10A	109.1
S3—C4—H4A	109.2	S6—C10—H10A	109.1
C3—C4—H4B	109.2	C9—C10—H10B	109.1
S3—C4—H4B	109.2	S6—C10—H10B	109.1
H4A—C4—H4B	107.9	H10A—C10—H10B	107.8
C6—C5—S3	114.9 (2)	C12—C11—S6	115.0 (2)
C6—C5—H5A	108.5	C12—C11—H11A	108.5
S3—C5—H5A	108.5	S6—C11—H11A	108.5
C6—C5—H5B	108.5	C12—C11—H11B	108.5
S3—C5—H5B	108.5	S6—C11—H11B	108.5
H5A—C5—H5B	107.5	H11A—C11—H11B	107.5
C5—C6—S1	111.6 (2)	C11—C12—S4	112.6 (2)
C5—C6—H6A	109.3	C11—C12—H12A	109.1
S1—C6—H6A	109.3	S4—C12—H12A	109.1
C5—C6—H6B	109.3	C11—C12—H12B	109.1
S1—C6—H6B	109.3	S4—C12—H12B	109.1
H6A—C6—H6B	108.0	H12A—C12—H12B	107.8

Symmetry codes: (i) −*x*+2, −*y*+1, −*z*+1; (ii) −*x*+1, −*y*+1, −*z*+2.
